# Biomarkers mesuared within 24 hours from the onset of septic shock and 28-day mortality

**DOI:** 10.1186/2197-425X-3-S1-A81

**Published:** 2015-10-01

**Authors:** MV de la Torre-Prados, A García-de la Torre, A Puerto-Morlán, E Cámara-Sola, P Nuevo-Ortega, T Tsvetanova Spasova, A Férnandez-Porcel, C Rueda-Molina, A García-Alcántara

**Affiliations:** Hospital Universitario Virgen de la Victoria, Department of Intensive Care Medicine, Málaga, Spain; Instituto de Investigación Biomédica de Málaga (IBIMA), Málaga, Spain; Puerto Real University Hospital, Clinical Chemistry Department, Puerto Real, Spain

## Introduction

Despite the remarkable advances in septic shock (SSh) management is still among the primary causes of death worldwide and there have been no significant changes in mortality over the last few decades.

## Objectives

Taking into account the importance of early identification and intervention in patients with SSh in relation to prognosis, we studied few biomarkers, measured within 24 hours from the onset of SSh, along with clinical score to compare their prognostic value for SSh and evaluate their The purpose of the study was to ascertain the prognostic value and evaluate their usefulness.

## Methods

We conducted a prospective cohort study of 100 consecutive critically-ill adult ICU patients on first-time admission with SSh according to the Surviving Sepsis Campaign during one year. Demographic data, severity score (APACHEII and SOFA) and laboratory data were recorded. The program used for the data processing and statistical analysis was SPSS 15.0®

## Results

Our study included 100 patients, 59% were men, the respiratory tract (48%) and abdomen (24%) were the two most common primary sites of infection. The median length of ICU stay was 8 days; and the 28-day mortality rate was 36%. Non-survivors were older and had significantly higher APACHE II and SOFA scores on ICU admission. The median MR-proADM and lactate levels were significantly higher in 28 day- non-survivors, however the number of platelets was significantly lower in this group,

**Table 1:** Distribution of biomarkers within 24 hours.

Biomarker	Overall population N = 100 Median (IQR)	Survivors (n = 64) Median (IQR)	Non-Survivors (n = 36) Median (IQR)	P*
Age	64 (54.25-71)	63 (49-69)	66.5 (59-72.75)	0.04
APACHE II	26 (21.5-29.5)	25 (19-29)	27 (22.75-33)	0.002
SOFA	10 (8-12)	10 (8-11)	12 (10-14)	< 0.001
MR-proADM (nmol/L)	1.71 (0.65-4.11)	1.06 (0.53-3.03)	2.84 (1.1-4.75)	0.002
PCT (ng/mL)	7.63 (1.48-24.04)	4.96 (1.07-23.31)	12.85 (4.23-35.52)	ns
CRP (mg/L)	222.15 (131-337.9)	182.5 (131.62-96.24)	249.08 (124-349.55)	ns
Lactate (mmol/L)	2.39 (1.68-3.96)	2.21 (1.62-3.38)	3.17 (1.96-4.56)	0.015
Platelets (x103/µL)	172.5 (106.5-276)	200(143.5-296)	125.65.2-202.5)	0.003
Cholesterol (mg/dL)	119.5 (81.5-153.5)	125.5 (91.75-161)	105 (67.75-135.75)	ns

*Mann-Whitney test; IQR= interquartile range, MR-proADM = Mid-regional pro-adrenomedullin; PCT = Procalcitonin; CRP = C-reactive protein). MR-proADM was the best predictor of 28-day mortality with the largest AUC (0.77; 95% CI: 0.7-0.89), followed by platelets (0.76; 95% CI: 0.67-0.85) and lactate (0.74; 95% CI: 0.64-0.83).

## Conclusions

The protein MR-proADM, lactate, platelets are strongly associated with accurate assessment of the risk of mortality in SSh patients, and similar to the APACHE II and SOFA score, when measured within the first 24 hours of onset of SS.

## References

[1] Pezzilli R (2012). Time course of proadrenomedullin in the early phase of septic shock. A comparative study with other proinflammatory proteins. Panminerva Med.

